# Recreational Water Illness and Injury Prevention Week — May 20–26, 2013

**Published:** 2013-05-17

May 20–26, 2013, marks the ninth annual Recreational Water Illness and Injury Prevention Week. This observance highlights easy and effective steps swimmers can take to reduce health and safety risks at swimming pools, hot tubs/spas, and other recreational water venues.

Recreational water illness (RWI) can result from ingesting, inhaling aerosols of, or having contact with contaminated water from pools, hot tubs/spas, water play areas, interactive fountains, lakes, rivers, or oceans. These illnesses also can be caused by chemicals in the water or chemicals that volatilize from the water and cause indoor air quality problems.

With the incidence of RWI outbreaks increasing, swimmers need to practice good swimmer hygiene (e.g., taking a pre-swim shower and not swimming when ill with diarrhea) to help protect themselves and other swimmers from pathogens. Poor swimmer hygiene leads to microbial contamination of water in recreational water venues and thus can increase risk for RWI (1).* Additional information on healthy swimming is available at http://www.cdc.gov/healthyswimming.

Public health agencies also have a role in preventing RWIs. In the United States, no federal agency regulates the design, construction, operation, and maintenance of public swimming pools and other public treated recreational water venues. All pool codes are independently written and enforced by state and/or local agencies. In 2005, local, state, and federal public health officials and representatives of the aquatic sector identified the variation in pool codes as a barrier to RWI prevention. Since 2007, CDC and the New York State Department of Health have spearheaded development of the Model Aquatic Health Code (MAHC). The MAHC is a set of science-based and best-practice guidelines to reduce the risk for RWI, drowning, and pool chemical–associated health events. The first draft edition of the MAHC, which integrates 14 individual modules revised to address the first round of public comments, will be available for final public comment this summer. The first official edition of the MAHC is expected to be released by the 2014 summer swim season. Additional information on the MAHC is available at http://www.cdc.gov/mahc.

Injuries and drownings also can occur in and around recreational water. Drowning is the leading cause of injury death among children aged 1–4 years. On average, 10 persons die from drowning each day, including two aged <15 years (2). Additional information on water safety is available at http://www.cdc.gov/homeandrecreationalsafety/water-safety/index.html.

## Footnotes

* Additional information at http://www.cdc.gov/healthywater/swimming/protection/triple-a-healthy-swimming.html.
